# Navigating Challenges in Mixed Malaria Infections: A Case of Mixed Plasmodium falciparum and Plasmodium vivax Infection in a Patient With Glucose-6-Phosphate Dehydrogenase (G6PD) Deficiency

**DOI:** 10.7759/cureus.92720

**Published:** 2025-09-19

**Authors:** Calvin Koh Sii Lau, Xin Yi Kek, Wee Fu Gan

**Affiliations:** 1 Internal Medicine, Hospital Melaka, Melaka, MYS; 2 Infectious Diseases, Hospital Melaka, Melaka, MYS

**Keywords:** g6pd deficiency, mixed malaria infection, nucleic acid amplification test, plasmodium falciparum infection, plasmodium vivax infection

## Abstract

Malaria continues to be a significant health issue in various regions worldwide, including Malaysia. It is a vector-borne disease transmitted primarily by *Anopheles* mosquitoes. Mixed malaria infections are rarely reported and often underestimated globally. Early diagnosis is essential for timely treatment, which improves outcomes and reduces morbidity. We report a case of imported mixed malaria infection in a patient with glucose-6-phosphate dehydrogenase deficiency, in whom *Plasmodium falciparum* and *Plasmodium vivax* were identified through malaria PCR. The patient’s condition improved after completing a full course of IV artesunate, oral Riamet (artemether/lumefantrine), and primaquine.

## Introduction

Malaria remains a major public health threat in many parts of the world, including Malaysia. Historically, malaria was endemic in several nontropical regions. Over the years, increasing deforestation, rapid and uncontrolled urban growth, and widespread human displacement have contributed to the emergence of malaria outbreaks in previously nonendemic areas.

According to the *World Malaria Report 2024* by the World Health Organization, there were 263 million malaria cases worldwide, with case numbers steadily increasing since 2020. The disease was responsible for 597,000 deaths in 2023, with the African region accounting for over half of global malaria mortality [1].

Malaysia has experienced a surge in zoonotic malaria cases caused by *Plasmodium knowlesi*, which accounted for 87.4% of global cases in 2023, followed by Thailand and Indonesia. *Plasmodium falciparum *is most prevalent on the African continent [1], whereas *Plasmodium vivax *is the most common malaria parasite in regions outside sub-Saharan Africa.

Mixed malaria infections have also been reported across different parts of the world, with the highest incidence in East and Southern Africa [1]. This is concerning because mixed infections not only increase disease severity and mortality but also complicate treatment. They can reduce the efficacy of antimalarial drugs, potentially drive drug resistance, and hinder surveillance and control efforts due to diagnostic challenges.

## Case presentation

A middle-aged man from Cameroon, Africa, who had been living in Malaysia for over 20 years and had a medical history of type 2 diabetes mellitus, hypertension, and lumbar degenerative disc disease, presented in June 2023 with a five-day history of fever, vomiting, headaches, and reduced oral intake. His symptoms followed a recent one-month trip to his home country, Cameroon, from April to May 2023, where he stayed in a malaria-endemic area.

On clinical examination, he was jaundiced with mild pallor but had no hepatosplenomegaly. Other systemic examinations were unremarkable. An urgent blood film for malaria parasites (BFMP) was performed and was positive for* P. falciparum*, with a sexual count of 0 parasites/µL of blood and an asexual count of 82,690 parasites/µL of blood. A full blood count revealed anemia and thrombocytopenia. At the same time, elevated direct bilirubin, increased LDH, and a high reticulocyte count concurrent with anemia indicated the possibility of hemolysis. Blood tests also showed evidence of organ involvement, including acute kidney injury and transaminitis (Table 1). Based on these findings, the patient was diagnosed with severe *P. falciparum *malaria. Further history revealed that he had experienced more than 20 episodes of malaria during his early years in Cameroon. Other investigations, including tests for dengue, leptospirosis, blood cultures, and viral screening (hepatitis B surface antigen, hepatitis C antibody, and HIV antigen/antibody test), were negative.

Glucose-6-phosphate dehydrogenase (G6PD) screening using the fluorescent spot test was performed. The G6PD quantitative assay showed an enzyme level of 3.82 U/g Hb, with enzyme activity of 43.2%, which indicated a moderate reduction (10-60%) consistent with G6PD deficiency. However, in the setting of acute hemolysis, G6PD levels may appear falsely elevated.

The patient was started urgently on IV artesunate before being transitioned to oral Riamet (artemether/lumefantrine) once he showed clinical improvement and was able to tolerate oral intake. In view of the G6PD deficiency and the sexual count of 0 on BFMP, single-dose primaquine administration was discussed but ultimately not given.

Despite completing the full course of Riamet, the patient remained unwell, complaining of fever, lethargy, dizziness, and abdominal discomfort. Malaria PCR testing revealed a mixed infection with *P. falciparum *and *P. vivax*. However, repeated serial BFMPs were negative for malaria parasites. In view of the mixed malaria species identified by PCR, the patient was initiated on oral primaquine to eradicate *P. vivax*. Given his underlying G6PD deficiency and the associated risk of hemolysis, a discussion was held with the patient and his family prior to administration. Primaquine was prescribed at 0.75 mg/kg weekly for a total of eight doses. The patient also received counseling on malaria prevention and the use of malaria prophylaxis medication for future travel.

He was observed in the ward for three days to monitor for potential hemolysis after the first dose of primaquine. Weekly blood investigations remained stable, with no evidence of hemolysis (Table 1).

**Table 1:** Summary of blood investigations ACT: artemisinin-based combination therapies; ALT: alanine transaminase; AST: aspartate transaminase; BFMP: blood film for malaria parasites; HCO₃: bicarbonate; LDH: lactate dehydrogenase; pCO₂: partial pressure of carbon dioxide; pO₂: partial pressure of oxygen

Parameter	Day 1	Day 5 after completion of oral ACT	Two weeks after primaquine was given	Reference range
Hemoglobin	10.4	10.4	10.5	13.0-17.0 g/L
White blood cells	4.2	6.8	6.3	4.0-10.0 × 10⁹/L
Platelets	65	441	267	150-410 × 10⁹/L
Reticulocytes	3.6	-	-	0.5-2.5%
Urea	10.5	5	3.3	3.2-8.2 mmol/L
Sodium	135	136	138	136-145 mmol/L
Potassium	3.9	4.3	4.2	3.5-5.1 mmol/L
Creatinine	109	77	81	62-115 µmol/L
Total bilirubin	152	39	19	<21.0 µmol/L
Direct bilirubin	116	-	-	<5.1 µmol/L
Indirect bilirubin	35.1	-	-	<15.9 µmol/L
Albumin	29	37	35	32-48 g/L
Globulin	37	39	37	25-39 g/L
ALT	55	38	38	10-49 U/L
AST	72	41	36	<34 U/L
LDH	599	265	258	120-246 U/L
pH	7.56	-	-	7.35-7.45
pCO₂	22	-	-	35-48 mmHg
pO₂	178	-	-	83-108 mmHg
HCO₃	19.7	-	-	18.0-23.0 mmol/L
Lactate	1.5	-	-	0.5-2.2 mmol/L
BFMP	*Plasmodium falciparum* (asexual count: 82,690; sexual count: 0)	No malaria parasites were seen after Day 2.		Parasite density >100,000/µL indicates severe malaria infection.

**Figure 1:**
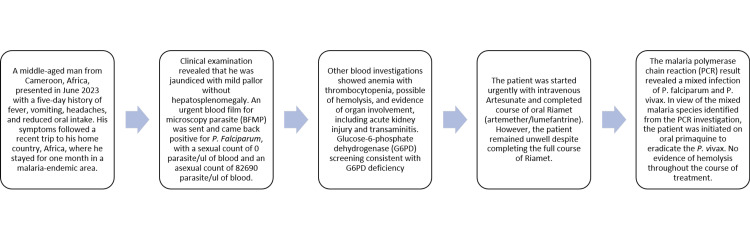
Summary of case presentation

A summary of the case presentation is provided in Figure 1. 

## Discussion

The five species of *Plasmodium* that cause human malaria are *P. falciparum*, *P. vivax*, *Plasmodium malariae*, *Plasmodium ovale*, and *Plasmodium knowlesi *[2]. Numerous studies worldwide have shown that mixed malaria infections are not uncommon, yet they are often underestimated and underrecognized [2,3]. However, such infections are relatively uncommon in Malaysia.

According to data from the CDC, *P. knowlesi *is the most prevalent malaria species in Malaysia, with most cases reported in Sabah and Sarawak due to its zoonotic mode of transmission [4]. In contrast, in African countries such as Cameroon, *P. falciparum *is the predominant species, followed by *P. malariae*, *P. ovale*, and *P. vivax *[1].

Accurate identification of mixed malaria infections is critical, as misdiagnosis can result in the development of severe disease and may contribute to antimalarial drug resistance [2]. Mixed malaria infections involving two or more *Plasmodium *species can be attributed to several factors (Table 2) [5].

**Table 2:** Factors attributed to mixed malaria infection Source: Escalante et al. (2022) [5]; Creative Commons Attribution (CC BY) license

Vector-related factors	Host-related factors	Parasite-related factors
Seven species of *Anopheles* shown to carry more than one species of *Plasmodium*	Residing in or traveling to endemic areas, inadequately treated previous infection, and poor adherence to therapy	Different timing of gametocyte production and maturation, different timing of hypnozoite formation, and different life span timing

In this patient, infection was most likely acquired during his recent travel home. This highlights the importance of obtaining a comprehensive travel history and maintaining a high index of suspicion in travelers from endemic regions. We postulated that he may have experienced multiple prior malaria infections that were inadequately treated, a factor contributing to mixed malaria infections. However, he was unable to recall his treatment history, including whether he had received prolonged daily or weekly courses of primaquine.

*Plasmodium* species have a complex life cycle involving both an intermediate vertebrate host and a definitive insect host. When an infected mosquito takes a blood meal, sporozoites from its salivary glands are inoculated into the skin, enter the bloodstream, and invade hepatocytes to escape host immunity and initiate asexual replication. Within several days, multinucleated exo-erythrocytic schizonts (meronts) develop. These rupture, releasing merozoites into the bloodstream that invade red blood cells. The sexual cycle begins when a subset of asexual parasites differentiate into gametocytes, which can circulate for several days, thereby maximizing their transmission to mosquitoes [6].

Mixed infections can occur through simultaneous inoculation. During the preerythrocytic stage, *P. falciparum *has a shorter developmental period than *P. vivax *(approximately 10 vs. 12 days), often leading to higher parasite loads of *P. falciparum *in mixed infections [7]. Although both species share similar erythrocytic development durations, *P. vivax *preferentially invades reticulocytes, whereas *P. falciparum* can invade erythrocytes of all ages [7]. This selective preference results in significantly lower parasitemia in *P. vivax *infections, which may be missed by microscopy as submicroscopic parasitemia.

BFMP remains the gold standard for diagnosis, severity assessment, and species identification [8]. However, diagnosing mixed infections is challenging because of species similarities under the microscope, low parasitemia, and the subjective nature of microscopy, which depends on the morphologist’s expertise. In such cases, nucleic acid amplification tests (NAATs) are valuable, as they can detect submicroscopic parasitemia and accurately identify species [4]. For example, a local study by Joveen-Neoh et al. in Sabah found misdiagnosis in 74 of 243 (30.5%) samples, with 24 microscopy-negative samples later identified as positive (including four mixed infections) using PCR [4].

Despite their sensitivity, NAATs are limited by turnaround time, availability, and cost [8]. They may not be routinely included in malaria eradication programs, but clinicians should consider them when patients remain symptomatic despite repeated negative BFMP results [8]. In this case, the initial BFMP was confirmed by the national malaria laboratory, but mixed infection was only identified through PCR. Reliance on BFMP alone could therefore miss mixed-species infections, with implications for both individual outcomes and public health. In this patient, concern also arose regarding the potential reintroduction of *P. vivax* into the local community, as he is a long-term resident of Malaysia through marriage.

Primaquine, an 8-aminoquinoline, is active against mature *P. falciparum *gametocytes, liver-stage parasites of all *Plasmodium *species, and dormant hypnozoites of *P. vivax *and *P. ovale *and has weak activity against asexual stages [9]. It should be used cautiously in patients with hemolytic anemia, G6PD deficiency, or a family/personal history of favism [9]. Primaquine can cause oxidant-mediated hemolytic anemia and methemoglobinemia, with severity depending on dose and degree of G6PD deficiency. During acute hemolysis, G6PD levels may appear falsely normal because older erythrocytes with low enzyme content are destroyed first, leaving younger cells with normal activity [10]. Thus, G6PD testing during acute hemolysis must be interpreted carefully. In this case, the patient’s haptoglobin level was markedly low (<0.0706 g/L; reference range, 0.3-2.0 g/L) when quantitative G6PD testing was performed.

## Conclusions

This case highlights the diagnostic and therapeutic challenges of mixed malaria infections, particularly in patients with travel to endemic regions and a history of G6PD deficiency. Identification of* P. vivax *coinfection through PCR underscores the limitations of conventional microscopy in low-parasitemia settings. These findings emphasize the need to integrate advanced molecular diagnostic techniques when patients remain symptomatic despite negative blood films. Earlier detection of mixed malaria infections through such methods can facilitate timely treatment and reduce the risk of local outbreaks in Malaysia.

## References

[1] (2024). World Malaria Report 2024. https://www.who.int/publications/i/item/9789240104440.

[2] Kotepui M, Kotepui KU, De Jesus Milanez G, Masangkay FR (2020). Plasmodium spp. mixed infection leading to severe malaria: a systematic review and meta-analysis. Sci Rep.

[3] Al-Subaie AM (2021). Case report: case report: mixed infection of Plasmodium vivax and Plasmodium falciparum in a tertiary hospital. F1000Res.

[4] Joveen-Neoh WF, Chong KL, Wong CM, Lau TY (2011). Incidence of malaria in the interior division of Sabah, Malaysian Borneo, based on nested PCR. J Parasitol Res.

[5] Escalante AA, Cepeda AS, Pacheco MA (2022). Why Plasmodium vivax and Plasmodium falciparum are so different? A tale of two clades and their species diversities. Malar J.

[6] Venugopal K, Hentzschel F, Valkiūnas G, Marti M (2020). Plasmodium asexual growth and sexual development in the haematopoietic niche of the host. Nat Rev Microbiol.

[7] Bourgard C, Albrecht L, Kayano AC, Sunnerhagen P, Costa FT (2018). Plasmodium vivax biology: insights provided by genomics, transcriptomics and proteomics. Front Cell Infect Microbiol.

[8] Mathison BA, Pritt BS (2017). Update on malaria diagnostics and test utilization. J Clin Microbiol.

[9] (2020). Assessment of long-term health effects of antimalarial drugs when used for prophylaxis. https://pubmed.ncbi.nlm.nih.gov/32369311/.

[10] Taylor WR, Kim S, Kheng S (2021). Dynamics of G6PD activity in patients receiving weekly primaquine for therapy of Plasmodium vivax malaria. PLoS Negl Trop Dis.

